# Supplementary material for: Unconscious reinforcement learning of hidden brain states supported by confidence
Source: Nat Commun. 2020 Aug 31;11:4429. doi: 10.1038/s41467-020-17828-8 (PMC7459278; doi:10.1038/s41467-020-17828-8)
Supplement: Supplementary file 1 — Supplementary Information [file 41467_2020_17828_MOESM1_ESM.pdf]

Supplementary Information

Unconscious reinforcement learning of hidden brain states supported by confidence

Aurelio Cortese, Hakwan Lau, Mitsuo Kawato

Supplementary Figures 1 – 11

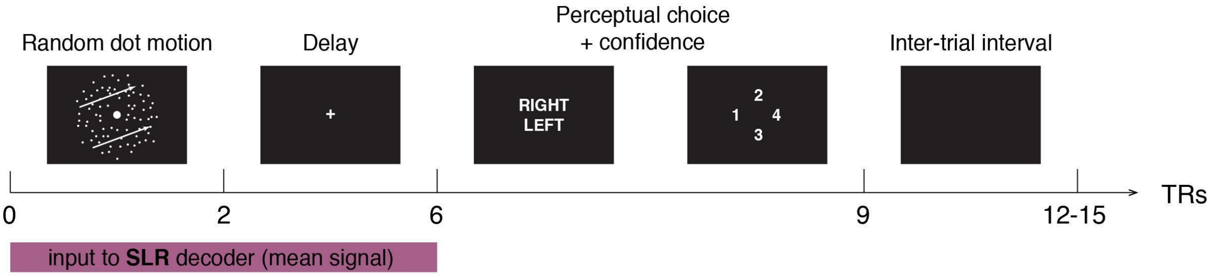

Supplementary Figure 1. **Session 0: motion discrimination task with confidence judgement for decoder construction**. About one week prior to the main RL experiment, subjects engaged in a two-choice direction discrimination task with confidence judgements while in the fMRI scanner. The purpose of this session was to construct subject-specific motion decoders. Each trial featured a random dot motion stimulus with high or low motion coherence presented for 2 sec, followed by a delay period of 4 sec. Subjects were then instructed to choose a motion direction (left or right), and indicate their confidence in their choice (1 to 4) by pressing a button on a response-pad corresponding to the indication presented on the screen. A trial ended with an inter-trial interval of variable length (3 to 6 seconds). For decoding, the 6 seconds (6 TRs) from onset of motion to end of delay period were used to train the decoders.

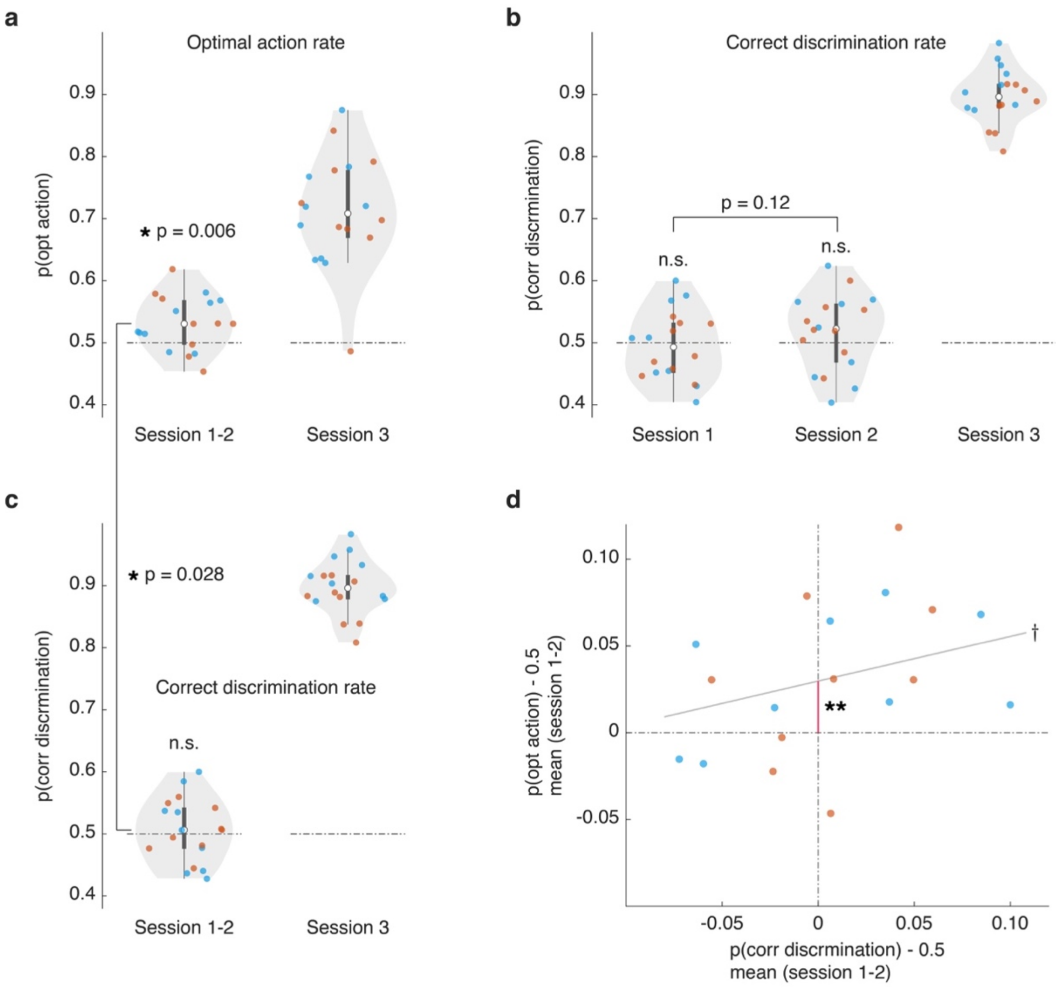

Supplementary Figure 2. **Optimal action rates, discrimination choices, and correlation. a** Optimal action rates averaged over sessions 1-2. **b** Subjects discrimination accuracies are plotted for each session. **c** Correct discrimination rates averaged over sessions 1-2. **d** Linear regression between optimal action rate and correct discrimination rate, both averaged over sessions 1-2. Intercept (**): *P*=0.0077, slope (†): *P*=0.089, (two-sided p-values). In **a**, **b**, **c** significance against chance was assessed with full linear models, with the intercept as difference from chance (two-sided p-values, uncorrected). The significance of the difference between two sessions (or average thereof) was assessed with sign tests (one-tailed p-value, uncorrected). The shaded areas in the violin plots represent the population spread and variance, the white dot at the centre the median, the thicker line the interquartile range. Coloured dots (orange: VC, blue: PFC) represent individual subjects, N = 18 biologically independent samples.

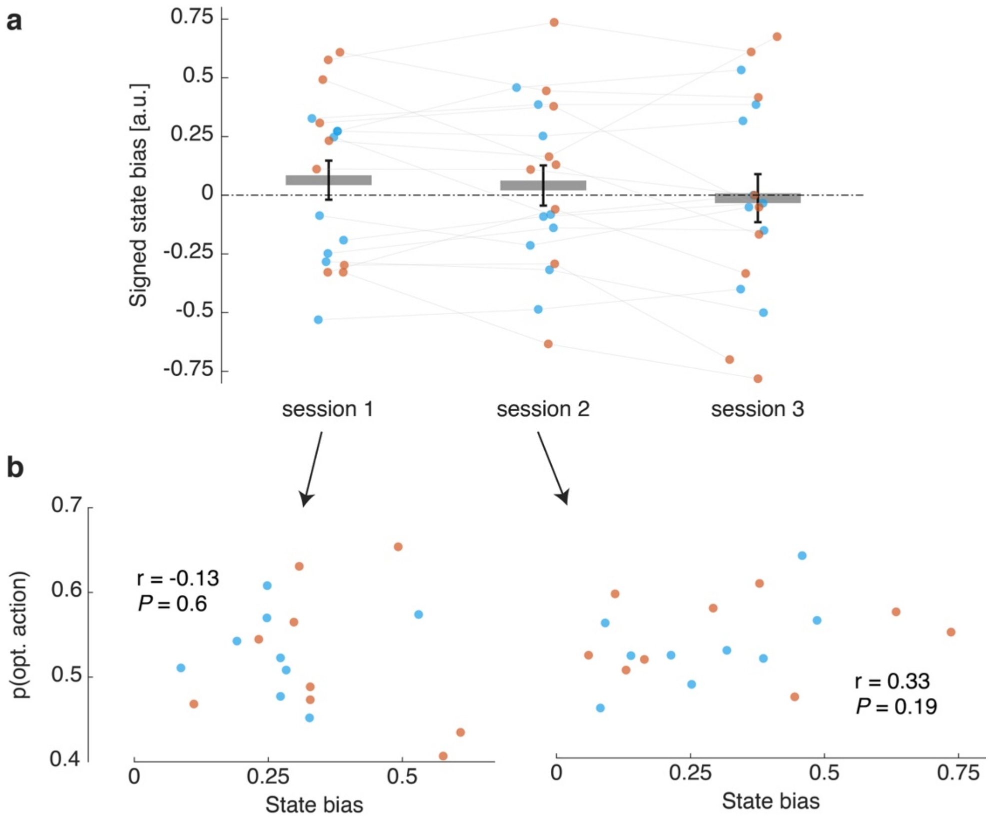

Supplementary Figure 3. **(Decoder) state-bias and relation to optimal action selection. a** Signed state-bias is plotted for each session. Negative values indicate a bias towards state 'left motion' while positive values indicate a bias towards state 'right motion'. Note that for most subjects the sign of the state-bias was constant between sessions 1 and 2 (17 no change, 1 change). Thick lines represent the median, error bars the SEM. **b** Pearson correlation (n = 18) between unsigned state-bias and optimal action rate, for each session, two-sided p-values. For all plots, coloured dots represent individual subjects, N = 18 biologically independent samples.

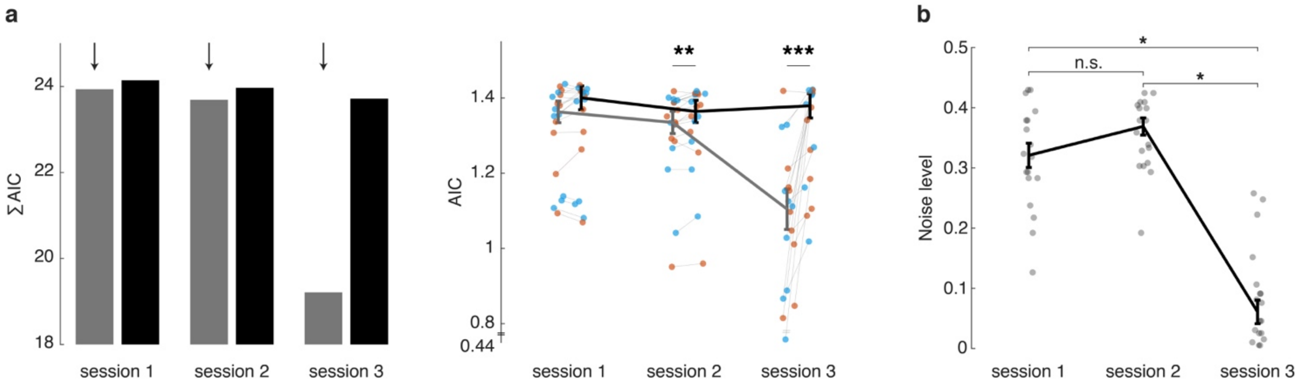

Supplementary Figure 4. **Normalized AIC and noise level for noisy state-dependent RL. a** AIC was computed using the sample-normalized log-likelihood, to bring AIC in the same absolute scale on each session. Left: bar graph shows the sum of AIC over the population; vertical arrows indicate the model with lower AIC. Right: AIC is plotted for each subject, and model, on each session. Error bars represent the SEM centred around the median, coloured dots individual subjects (orange = VC, light blue = PFC). Significance of the AIC difference with sessions was assessed with sign tests (two-sided p-values, FDR corrected): **(session2) $P = 0.01$, ***(session 3) $P < 0.001$. **b** The noise level was estimated by maximizing the log-likelihood of the RL_{sd} model. For each subject and session, 100 resampling runs were tested, with noise ranging from 0 to 0.5, with 100 steps. The optimal noise level was selected between 0 and 0.5 leading to the highest log-likelihood. Grey dots represent individual subjects, error bars the SEM centred around the median. Statistical comparisons were computed with Wilcoxon signed-rank test (two-tailed p-values, FDR corrected): n.s.(session 1-2) $P = 0.34$, *(session 1-3) $P < 0.001$, *(session 2-3) $P < 0.001$. For all plots with individual data displayed, N = 18 biologically independent samples. ** $P < 0.01$, *** $P < 0.005$.

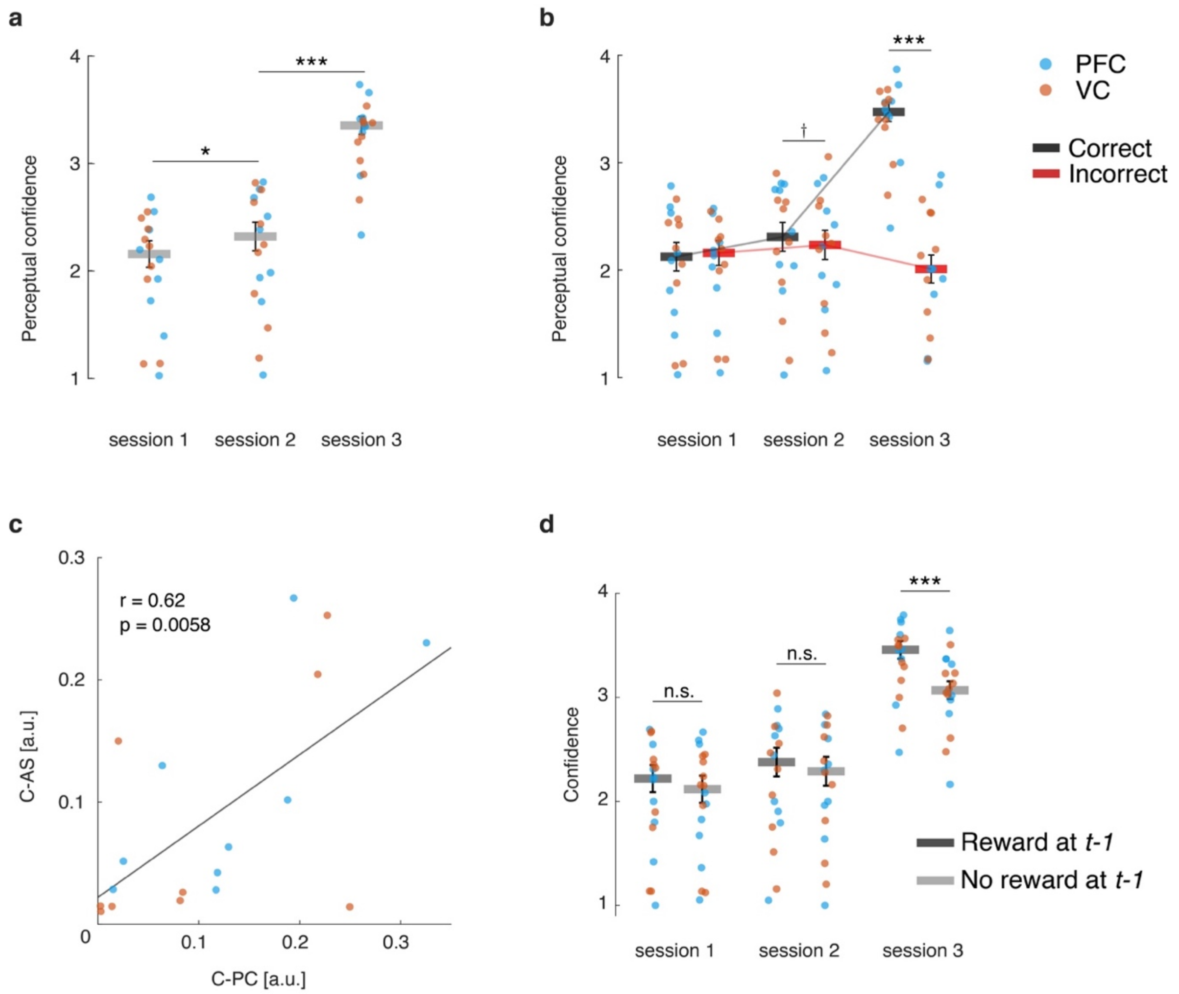

Supplementary Figure 5. **Analysis of behaviour: confidence ratings. a** Confidence judgments for each session. Between-session statistical test of difference: Wilcoxon signed-rank test (one-sided p-values, uncorrected). **b** Confidence judgements for correct and incorrect trials (discrimination, based on decoder output). Within-session statistical test of difference (correct vs error): Wilcoxon signed-rank test (one-sided p-values, uncorrected). **c** Overall strength of confidence effect in action-selection and perceptual discrimination were correlated in session 2. C-PC: measure of confidence effect on perceptual choice; C-AS: measure of confidence effect on action-selection. The measure was computed as the magnitude of the averaged (signed) differences in performance over confidence levels. The grey line represents a least square fit. Pearson correlation (n = 18), two-sided p-value. **d** Confidence judgments were divided according to the action selection outcome at *t-1*. Within-session statistical test of difference (Reward vs no reward): Wilcoxon signed-rank test (two-sided p-values, uncorrected). For all plots, coloured dots represent individual subjects, N = 18 biologically independent samples. In **a**, **b**, **d**: thick lines represent the median, error bars the SEM. **a**: *(session 1-2) *P* = 0.0277, ***(session 2-3) *P* = 0.0002, **b**: †(session 2) *P* = 0.06, ***(session 3) *P* = 0.0002, **d**: n.s.(session 1) *P* = 0.25, n.s.(session 2) *P* = 0.27, ***(session 3) *P* = 0.005.

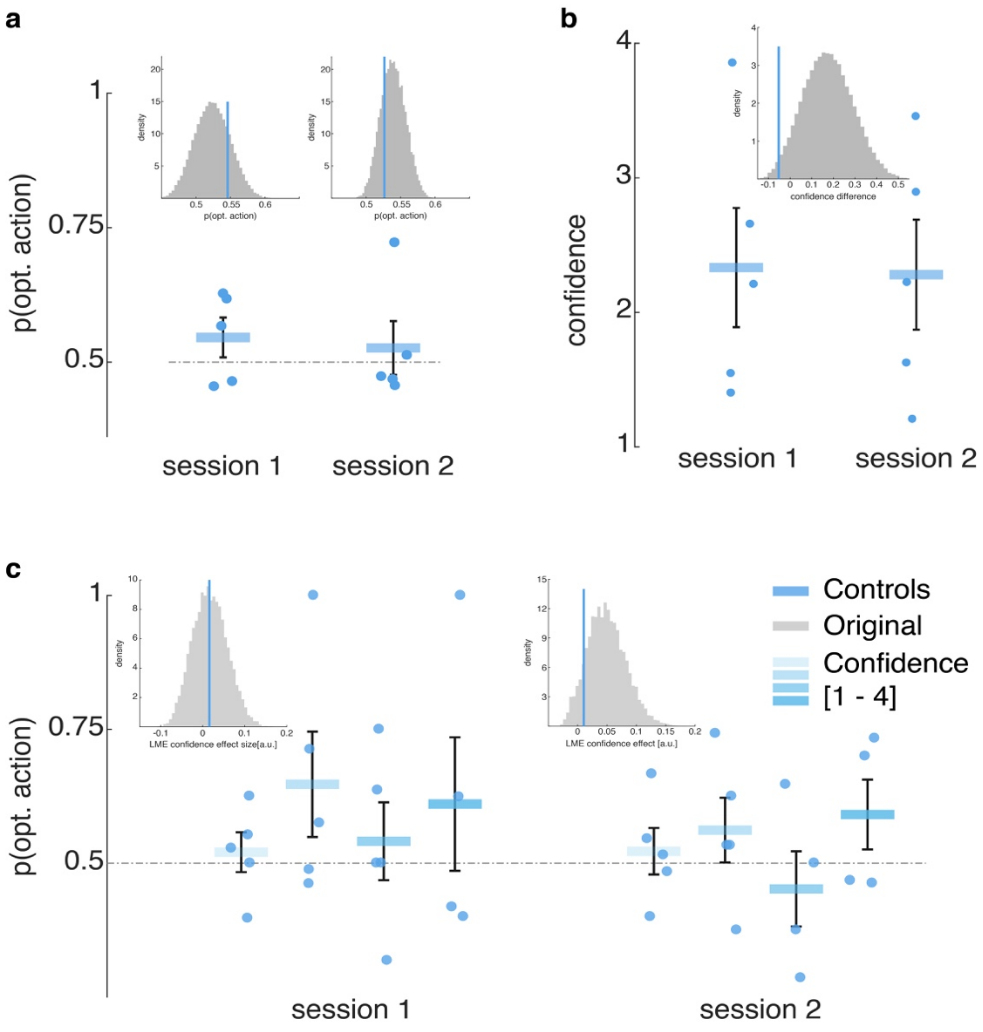

Supplementary Figure 6. **Control experiment: action-selection and confidence in naive subjects without closed-loop**. The same RL task was submitted to naive subjects. As in the main experiment, physical stimuli had zero coherence. Furthermore, trials were determined as yoked sequences from those of subjects who did the full closed-loop experiment. **a** Optimal action-selection for session 1 and session 2. Inset histograms depict the distributions (session 1, session 2) of all possible random draws of N = 5 from the original subjects' data. The coloured vertical line represents the average of N = 5 control subjects. **b** Mean confidence ratings in session 1 and session 2. Inset histogram depicts the distribution of confidence difference between session 2 and session 1 of all possible random draws of N = 5 from the original subjects' data. The coloured vertical line represents the average of N = 5 control subjects. **c** Optimal action-selection subdivided by confidence level. Inset histograms depict the effect size of the confidence effect, as measured with linear mixed effect model, for each session parametrized as *y ~ confidence + (1 | subjects)*, using all possible combinations of N = 5 from the original subjects' data. The coloured vertical line represents the average of N = 5 control subjects. For all plots, coloured dots represent individual subjects, thick lines the mean, error bars the SEM, N = 5 biologically independent samples.

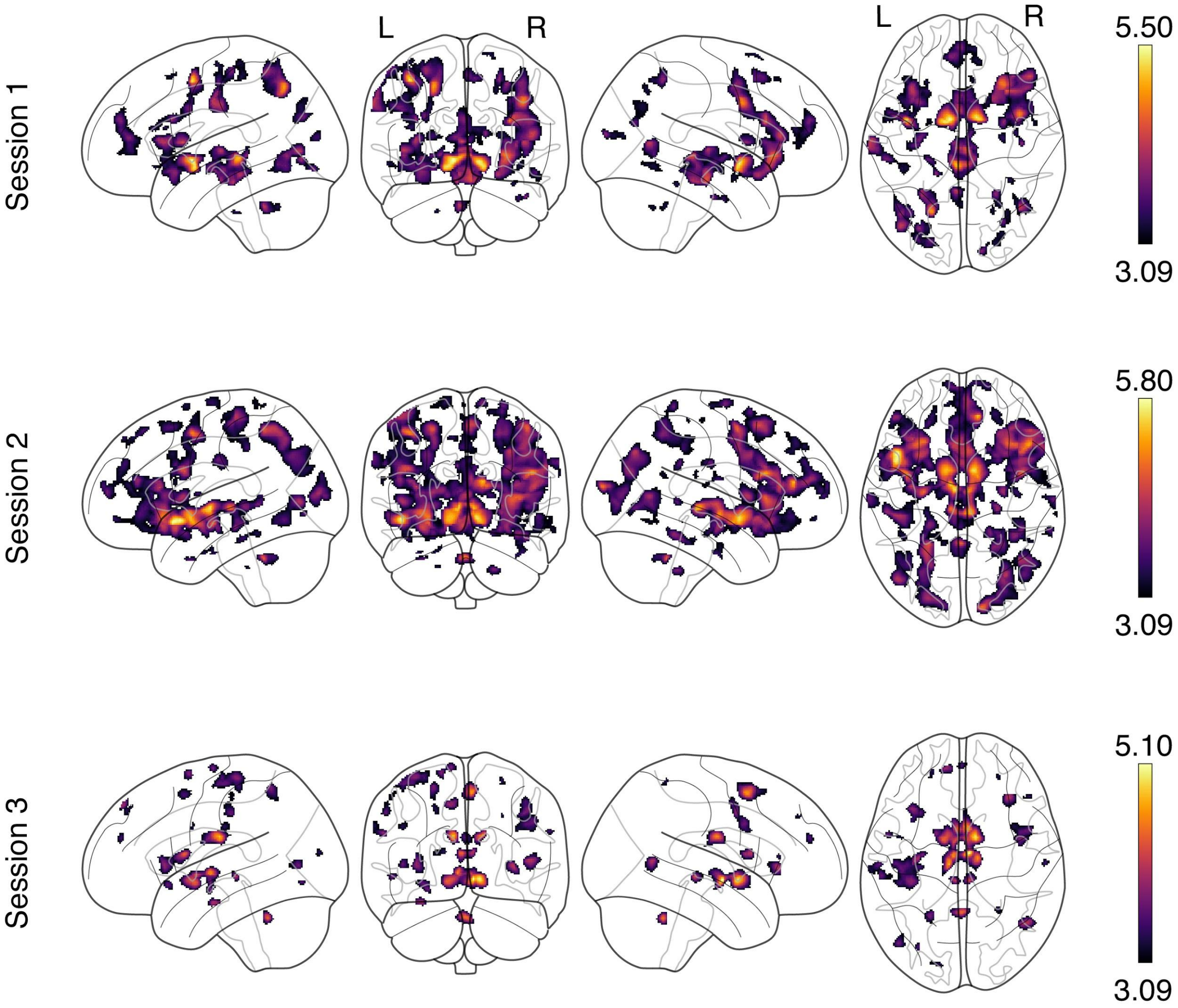

Supplementary Figure 7**: Neural correlates of raw RPE.** Glass brain plots of signed RPE correlates from the RL_{sd} model for each session. Statistical parametric maps were generated with a general linear model with RPE as a parametric regressor**.** Group-level statistical parametric maps are plotted at $Z > 3.09$, $p(FPR) < 0.001$ (false positive control meaning of cluster forming), cluster size $k > 30$.

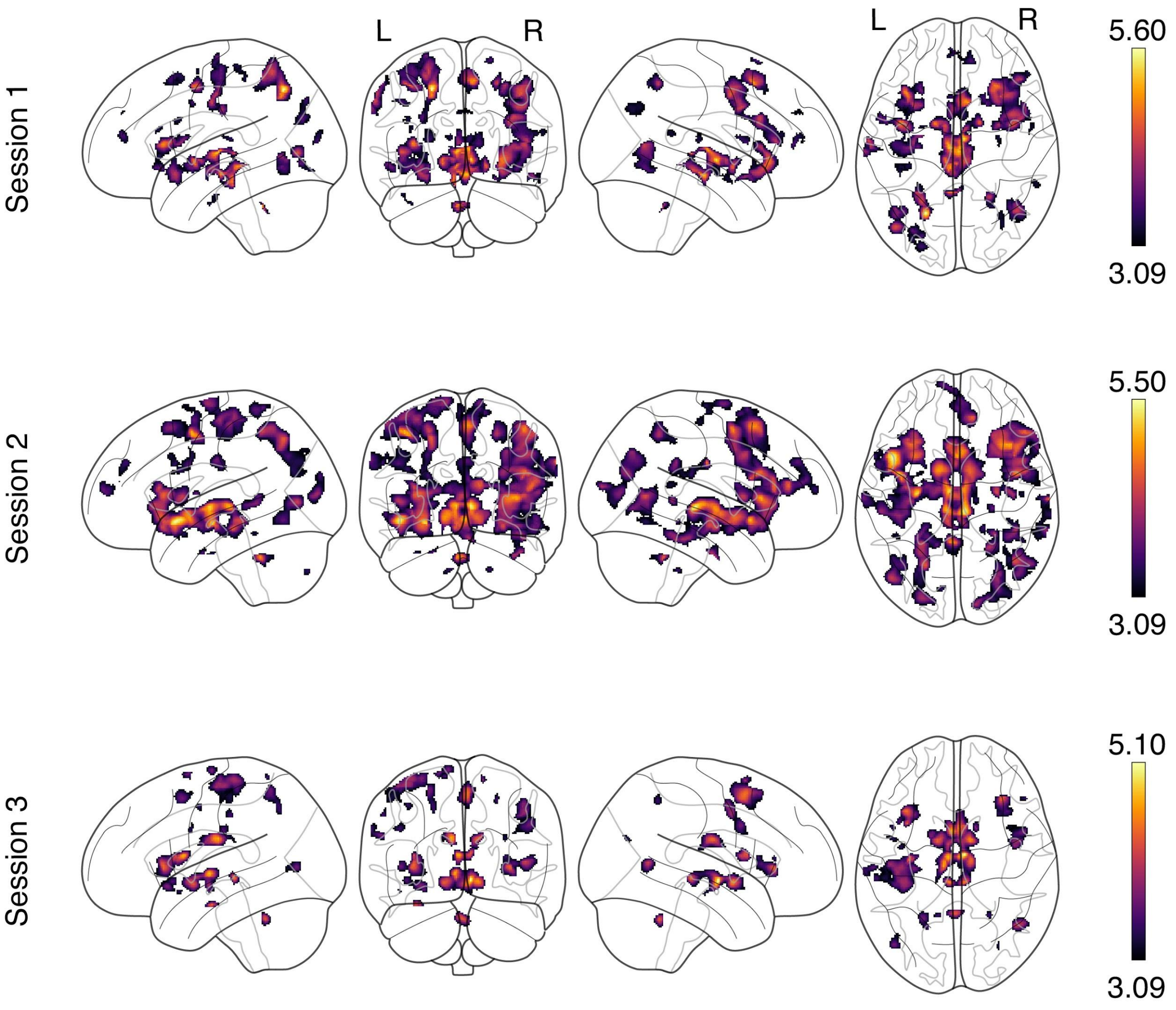

Supplementary Figure 8**: Neural correlates of z-scored RPE.** Glass brain plots of z-scored (across subjects and sessions) RPE correlates from the RL_{sd} model for each session. Statistical parametric maps were generated with a general linear model with RPE as a parametric regressor**.** Group-level statistical parametric maps are plotted at $Z > 3.09$, $p(FPR) < 0.001$ (false positive control meaning of cluster forming), cluster size $k > 30$.

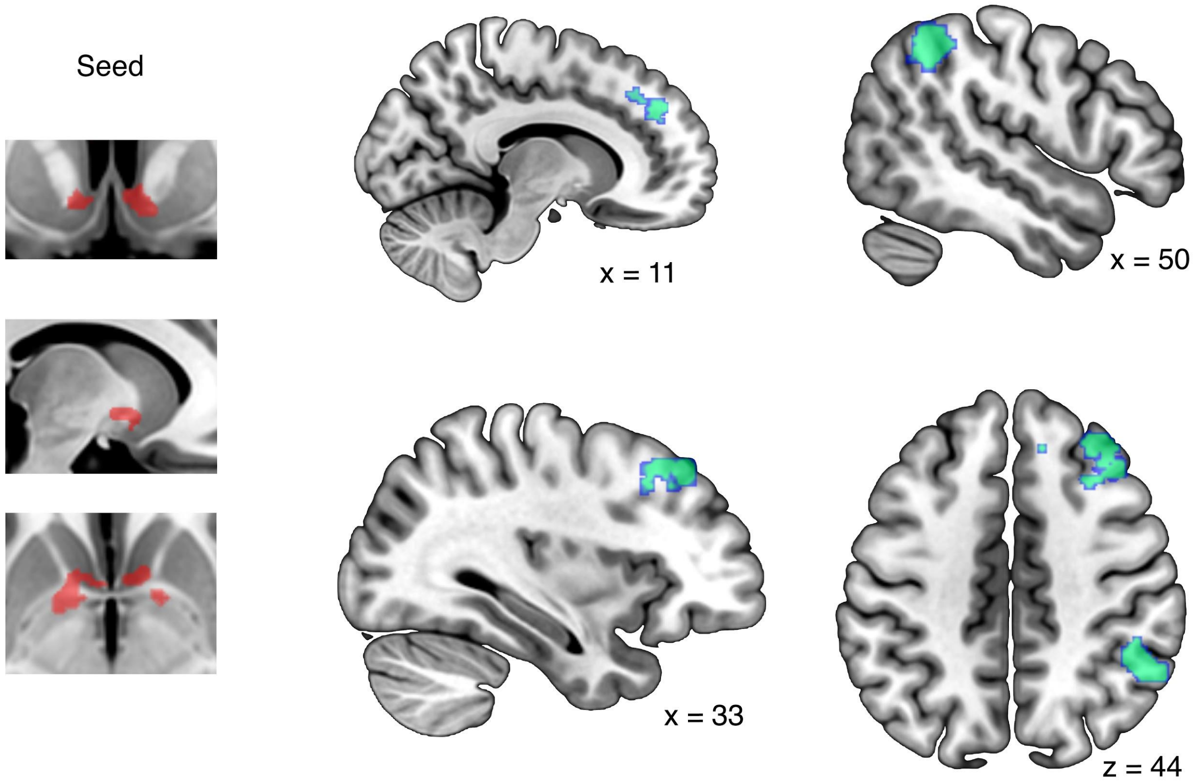

Supplementary Figure 9. **Functional connectivity analysis**. The seed region in the basal ganglia was defined from the RPE analysis of session 3 - independent data, collected after the last resting-state scan (defined at $p<0.001$, within an anatomical mask of the basal ganglia). The analysis was restricted to the resting state scan before and after Session 2, because this session was the single time point where subjects showed evidence of learning, but where the RL states were still latent, unconscious. Group-level statistical parametric maps plotted at p(unc.) < 0.001 (height: $t > 3.65$), and p(FDR) < 0.05 (cluster size). The map was created by applying the contrast [-1 1] over the 2nd and 3rd resting-state scans (before and after session 2, to capture changes related to that particular session), one-sided to test for increases in connectivity. No voxels survived for decrease in connectivity under the same conditions.

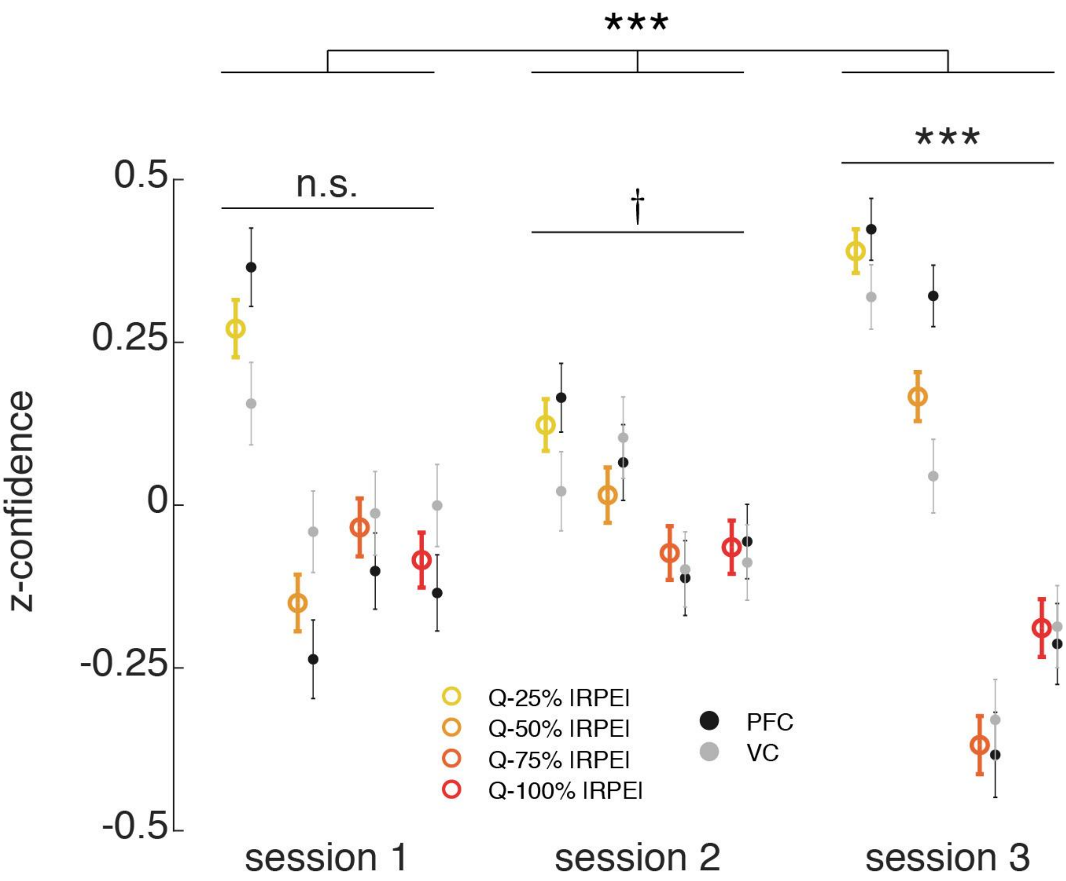

Supplementary Figure 10. **Computational modelling of behaviour: effects of reinforcement learning at trial *t* on confidence at trial *t+1*.** |RPE| (the magnitude of RPE) modulation of confidence. |RPE| data from each session were binned into quartiles. The mean z-scored confidence (computed over the whole session data, for display purposes only) was then plotted for each bin. Statistical significance was evaluated with linear mixed effects models (two-sided p-values). Coloured circles represent the median across all subjects pooled, light grey circles represent the median across subjects pooled from the VC group, and dark from the PFC group; error bars the SEM. N = 18 biologically independent samples. **(interaction) $P < 10^{-3}$, †(session 2) $P = 0.084$, ***(session 3) $P < 10^{-3}$

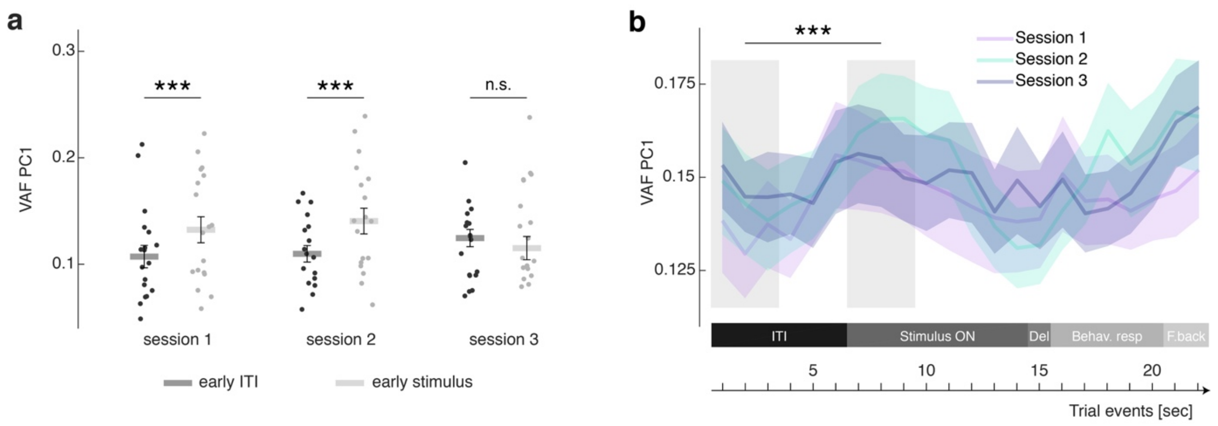

Supplementary Figure 11. **Functional dimensionality of activity patterns in target ROI.** Voxels' activity patterns from the ROI used for online decoding were input into a PCA. The variance-accounted-for (VAF) of the first PC was taken as indexing the functional dimensionality. Higher VAF indicates that the neural activity patterns have lower functional dimensionality. **a** VAF for the first PC, using mean activity patterns over the first 3TRs of the early inter-trial interval (ITI) and stimulus periods. Plots for each session, left, dark grey data represent the 3 TRs from early ITI; right, light grey data represent the 3 TRS from stimulus onset. Coloured dots represent individual subjects, thick line the median, and error bars the SEM. Statistical significance of the difference in VAF between early ITI and early stimulus was evaluated with Wilcoxon signed-rank test (two-sided p-values, FDR-corrected). ***(session 1) $P = 0.0024$, ***(session 2) $P = 0.0018$, n.s.(session 3) $P = 0.18$. **b** Same analysis as in **a**, but computed with data from each TR. Coloured lines represent mean data from all subjects, for each session and TR, shaded areas the SEM. Statistical significance was evaluated with linear mixed effects models (two-sided p-values), full results are reported in table ST7. N = 18 biologically independent samples. *** $P < 0.005$.

Supplementary Tables 1 – 9

**y = probability of selecting optimal action or probability of correct discrimination**				
**formula (reduced model)**		*y ~ 1 + tasktype + session + (1|subjects)*		
**Likelihood ratio test (reduced vs. full model)**		LRStat = 0.089 (*P* = 0.77)	deltaDF = 1	
	**β (SE)**	**tStat**	**DF**	***P***
task type	**-0.026 (0.011)**	**-2.24**	**69**	**0.028 ***
session	0.019 (0.011)	1.63	69	0.11
**Random effects**		**Name**		**Estimate**
	subjects (18)	intercept (std)		0.03
	error	sqrt (dispersion)		0.0485

Supplementary Table 1. **LME model - testing for difference between task type (action selection vs discrimination) on probability of correct action/choice.** Significance based on two-sided p-values.

**y = probability of selecting optimal action**				
**formula (reduced model)**		*y ~ 1 + tasktype + session + (1|subjects)*		
**Likelihood ratio test (reduced vs. full model)**		LRStat = 0.089 (*P* = 0.77)	deltaDF = 1	
	**β (SE)**	**tStat**	**DF**	***P***
pd_{t-1}	0.002 (0.015)	0.158	4375	0.87
**Random effects**		**Name**		**Estimate**
	subjects (18)	intercept (std)		0.126
	session (2)	intercept (std)		0.016
	error	sqrt (dispersion)		0.483

Supplementary Table 2. **LME model - effect of perceptual discrimination choice in previous trial on the probability of selecting optimal action.** Significance based on two-sided p-values.

**y = probability of optimal action**				
**formula**	*y ~ 1 + group*session + group*confidence + session*confidence + (1|subjects)*			
**Likelihood ratio test (reduced vs. full model)**	LRStat = 0.089 (*P* = 0.77); deltaDF = 1			
**Fixed effects**	**β (SE)**	**tStat**	**DF**	***P***
group	-0.050 (0.086)	-0.608	194	0.544
session	-0.052 (0.083)	-1.399	194	0.163
confidence	-0.041 (0.037)	-1.402	194	0.163
group:session	-0.028 (0.029)	-0.993	194	0.322
group:confidence	0.033 (0.028)	1.529	194	0.128
session:confidence	**0.041 (0.021)**	**3.179**	**194**	**0.0017 ****
**Random effects**		**Name**		**Estimate**
	subjects (18)	intercept (std)		0.054
	error	sqrt (dispersion)		0.16

**Session 1 [***y ~ 1 + confidence + group + (1|subjects)***]**				
group	0.006 (0.055)	0.11	62	0.91
confidence	0.018 (0.020)	0.87	62	0.39
Random effects:	subjects (18)	intercept (std)		0.071
	error	sqrt (dispersion)		0.18
**Session 2 [***y ~ 1 + confidence + group + (1|subjects)***]**				
group	-0.027 (0.039)	-0.70	62	0.49
confidence	**0.047 (0.016)**	**2.98**	**62**	**0.004 ****
Random effects:	subjects (18)	intercept (std)		0.040
	error	sqrt (dispersion)		0.14
**Session 3 [***y ~ 1 + confidence + group + (1|subjects)***]**				
group	-0.051 (0.049)	-1.04	68	0.30

confidence	**0.10 (0.018)**	**5.57**	**68**	**$<10^{-3}$** ***
Random effects:	subjects (18)	intercept (std)		0.057
	error	sqrt (dispersion)		0.17

Supplementary Table 3. **LME model - effect of confidence on the probability of optimal action-selection.** Significance based on two-sided p-values.

y = probability of correct discrimination				
**formula**	*y ~ 1 + group*session + group*confidence + session*confidence + (1|subjects)*			
**Likelihood ratio test (reduced vs. full model)**	LRStat = 1.02 (*P* = 0.32); deltaDF = 1			
**Fixed effects**	**β (SE)**	**tStat**	**DF**	***P***
group	0.019 (0.090)	0.207	194	0.840
session	-0.038 (0.043)	-0.879	194	0.381
confidence	-0.058 (0.034)	-1.718	194	0.087
group:session	0.027 (0.032)	0.824	194	0.411
group:confidence	-0.010 (0.024)	-0.426	194	0.67
session:confidence	**0.069 (0.015)**	**4.711**	**194**	**$<10^{-3}$ *****
**Random effects**		**Name**		**Estimate**
	subjects (18)	intercept (std)		0.002
	error	sqrt (dispersion)		0.19

**Session 1** [*y ~ 1 + confidence + group + (1|subjects)*]				
group	0.039 (0.047)	0.82	62	0.41
confidence	0.030 (0.022)	1.37	62	0.18
Random effects:	subjects (18)	intercept (std)		$< 10^{-3}$
	error	sqrt (dispersion)		0.19
**Session 2** [*y ~ 1 + confidence + group + (1|subjects)*]				
group	0.023 (0.051)	0.45	62	0.66
confidence	0.033 (0.018)	1.79	62	0.078 +
Random effects:	subjects (18)	intercept (std)		0.065
	error	sqrt (dispersion)		0.16
**Session 3** [*y ~ 1 + confidence + group + (1|subjects)*]				
group	0.081 (0.049)	1.67	68	0.10

confidence	**0.16 (0.017)**	**9.38**	**68**	**$< 10^{-3}$ *** **
Random effects:	subjects (18)	intercept (std)		0.063
	error	sqrt (dispersion)		0.16

Supplementary Table 4. **LME model - effect of confidence on the probability of correct discrimination.** Significance based on two-sided p-values.

**y = |RPE_{sd}| (|RPE| from RL_{sd})**				
**formula:** *y ~ 1 + group*session*confidence + (1|subjects)*				
**Fixed effects**	**β (SE)**	**tStat**	**DF**	***P***
group	0.0092 (0.039)	0.23	6649	0.81
session	**0.019 (0.008)**	**2.43**	**6649**	**0.015 ***
confidence	-0.0006 (0.007)	-0.10	6649	0.92
group:session	-0.0077 (0.011)	-0.67	6649	0.50
group:confidence	**0.028 (0.0096)**	**2.89**	**6649**	**0.004 *****
session:confidence	-0.0022 (0.0029)	-0.75	6649	0.45
group:session:confidence	**0.0095 (0.0041)**	**2.32**	**6649**	**0.02 ***
**Random effects**		**Name**		**Estimate**
	subjects (18)	intercept (std)		0.067
	error	sqrt (dispersion)		0.132

**Session 1 [***y ~ 1 + confidence + group + (1|subjects)***]**				
group	-0.029 (0.064)	-0.46	2059	0.64
confidence	0.001 (0.002)	0.41	2059	0.68
Random effects:	subjects (18)	intercept (std)		0.13
	error	sqrt (dispersion)		0.089
**Session 2 [***y ~ 1 + confidence + group + (1|subjects)***]**				
group	-0.039 (0.055)	-0.71	2348	0.48
confidence	**-0.0067 (0.0027)**	**-2.51**	**2348**	**0.012 ***
Random effects:	subjects (18)	intercept (std)		0.12
	error	sqrt (dispersion)		0.10
**Session 3 [***y ~ 1 + confidence + group + (1|subjects)***]**				
group	-0.0048 (0.017)	-0.28	2241	0.78
confidence	**-0.019 (0.0027)**	**-7.11**	**2241**	**$< 10^{-3}$ *****

Random effects:	subjects (18)	intercept (std)	0.035
	error	sqrt (dispersion)	0.12

Supplementary Table 5. **LME model - effect of confidence on |RPE| from RL_{sd}.** Significance based on two-sided p-values.

**y = confidence$_{t+1}$**				
**formula:** *y ~ 1 + group*session + group*|RPE| + session*|RPE| + (1|subjects)*				
**Likelihood ratio test (reduced vs. full model)**	LRStat = 0.04 (*P* = 0.84); deltaDF = 1			
**Fixed effects**	**β (SE)**	**tStat**	**DF**	***P***
group	-0.070 (0.20)	-0.35	6696	0.73
session	**0.73 (0.05)**	**15.72**	**6596**	**< 10^{-3} *****
|RPE|	**0.53 (0.23)**	**2.25**	**6596**	**0.024 ***
group:session	**0.088 (0.028)**	**3.15**	**6596**	**0.002 *****
group:|RPE|	-0.25 (0.17)	-1.46	6596	0.15
session:|RPE|	**-0.36 (0.10)**	**-3.50**	**6596**	**< 10^{-3}**
**Random effects**		**Name**		**Estimate**
	subjects (18)	intercept (std)		0.38
	error	sqrt (dispersion)		0.90

**Session 1 [***y ~ 1 + |RPE| + group + (1|subjects)***]**				
group	-0.014 (0.250)	-0.057	2041	0.95
|RPE|	0.30 (0.19)	1.57	2041	0.12
Random effects:	subjects (18)	intercept (std)		0.52
	error	sqrt (dispersion)		0.79
**Session 2 [***y ~ 1 + |RPE| + group + (1|subjects)***]**				
group	0.024 (0.261)	0.09	2330	0.93
|RPE|	-0.28 (0.16)	-1.73	2330	0.084 **+**
Random effects:	subjects (18)	intercept (std)		0.55
	error	sqrt (dispersion)		0.79
**Session 3 [***y ~ 1 + |RPE| + group + (1|subjects)***]**				
group	0.077 (0.15)	0.51	2223	0.61
|RPE|	**-1.11 (0.16)**	**-6.92**	**2223**	**< 10^{-3} *****

Random effects:	subjects (18)	intercept (std)	0.31
	error	sqrt (dispersion)	0.90

Supplementary Table 6. **LME model - effect of |RPE| from RL_{sd} on confidence at trial *t+1*.** Significance based on two-sided p-values.

**y = variance accounted for by PC-1**				
**formula:** *y ~ 1 + session*EL + (1|subjects)*				
**Likelihood ratio test (reduced vs. full model)**	LRStat = 2.55 (*P* = 0.11); deltaDF = 1			
**Fixed effects**	**β(SE)**	**tStat**	**DF**	***P***
session	0.001 (0.002)	0.75	321	0.45
EL	**0.019 (0.003)**	**6.38**	**321**	**$< 10^{-3}$ *****
**Random effects**		**Name**		**Estimate**
	subjects (18)	intercept (std)		0.039
	error	sqrt (dispersion)		0.027

Supplementary Table 7. **LME model - testing for difference in functional dimensionality differences.** The model compares the 3 times points in early pre-stimulus period (used for decoding to determine latent RL states) vs. the first 3 time points of early stimulus presentation (pure noise). Significance based on two-sided p-values.

Subject #	Brain region	Subregion decoder
S1	PFC	DLPFC
S2	VC	V1/2
S3	PFC	DLPFC
S4	VC	V1/2
S5	PFC	MFG
S6	VC	V1
S7	VC	V1/2/3
S8	PFC	MFS
S9	PFC	MFG
S10	VC	V1/2
S11	PFC	IFS
S12	VC	V1/2
S13	PFC	DLPFC
S14	VC	V1/2
S15	VC	V1/2/3
S16	PFC	MFG
S17	VC	V1/2/3
S18	PFC	MFG

Supplementary Table 8. **Subject-specific subregions selected for motion decoder**. Each subject was assigned to the PFC or VC group so as to minimize the intergroup difference in decoding accuracy (which could otherwise lead to a large confounding factor). Furthermore, within each region, the decoder based on the subregion that yielded the highest mean accuracy, with the lowest difference between the two classes (i.e., leftward vs. rightward motion), was selected. The table reports for each subject the group they were assigned to (VC or PFC) as well as their individual decoder used in the stage 2 online training. PFC: prefrontal cortex, VC: visual cortex, MFG: middle frontal gyrus, MFS: middle frontal sulcus, IFS: inferior frontal cortex, DLPFC: dorsolateral prefrontal cortex (combination of IFS, MFS, and MFG), V1: area V1 of VC, V1/2: areas V1 and V2 of VC, V1/2/3: areas V1, V2, and V3 of VC.

		session 1	session 2	session 3
State-dependent RL_{sd}	α	0.135 ± 0.069	0.112 ± 0.052	0.035 ± 0.009
	β	0.828 ± 0.302	0.741 ± 0.222	4.771 ± 1.035
State-free RL_{sf}	α	0.171 ± 0.066	0.113 ± 0.053	0.108 ± 0.041
	β	2.549 ± 0.815	2.288 ± 0.532	2.092 ± 0.491

Supplementary Table 9. **Estimated hyperparameters for RL_{sd} and RL_{sf}.** The models were fitted on individual data, on each session. Values represent group means ± SEM of each hyperparameter at the minimum log-likelihood.

Supplementary Notes (1 – 2)

(1)

By design, mental imagery or illusory perception during stimulus presentation could not directly affect the task since decoding was performed with data from pre-stimulus time points (although we used the first 3 sec from stimulus presentation to infer the latent RL state, after accounting for some hemodynamic delay, data points in fact coincide with pre-stimulus ITI). Furthermore, all subjects reported resting during the pre-stimulus period. At the end of each session subjects also reported their conscious strategies in selecting actions. These mentioned several possibilities, e.g., looking for patterns in the random dots. With time, subjects increasingly reported pairing action-selection with their perceptual discrimination choices, and always did so towards the end of the third session, indicating the emergence of a conscious understanding of the rule. Nota bene, reward contingencies were defined by the online decoder output, not discrimination choices. We also considered whether subjects could have used the previous trial's motion judgement to select an action. Brain activity related to the discrimination choice in the previous trial could have lingered long enough to be residually picked by the decoder, biasing its output towards either class. A regression analysis restricted to the first two sessions did not support this possibility (LME model, fixed effect 'perceptual choice at t-1' $\beta = 0.002$, $t_{4375} = 0.158$, $P = 0.87$, Supplementary Table 2).

(2)

Although based on stochastic representations captured by our decoders, one could always argue that, in principle, the problem is simple. For example, since the closed-loop learning task involved presentation of random dot-motion and a direction discrimination choice, subjects were already cued towards the latent state. Furthermore, subjective experience of random motion could have produced a specific direction representation. Or subjects could have figured to simply imagine motion direction, which in either case the decoder could have picked to define the latent RL state. Although plausible, these interpretations are unlikely in light of decoding effectively taking place during ITI when accounting for even short hemodynamic delays, and perceptual discrimination choices having very low congruency with respect to the decoder output. Subjects' belief was that the task depended on the motion direction they saw - not the arbitrary motion direction information that we inferred in real-time from patterns of brain activity. As such, without knowing that decoding takes place, that the critical window is in the pre-stimulus period, this remains a difficult and multidimensional problem for the brain. We confirmed that neural activity patterns in the early

ITI had higher functional dimensionality than in the early stimulus period (Supplementary Figure 11 and Supplementary Table 7). Even with implicit prior knowledge that a representation of motion direction is the relevant state, it should not be an obvious problem for the brain to figure out the spatial localization (PFC or VC) or sparsity of about 100 voxels selected by the machine learning algorithm and used in the RL sessions. Additionally, the original decoding accuracy of motion direction was around 70-75%. Due to noise, sometimes similar patterns of activity may be categorized as two different states. Since rewards are scheduled upon the decoder output, the presence of this decoding noise means that the original reward probabilities (0.8/0.2) are more likely to be in the range of 0.6/0.4, further complicating the problem. However, given the inherent limitations of these arguments, we acknowledge that probably the best way to resolve this question objectively would be to use a decoder with an arbitrary hyperplane boundary, where the classes do not correspond to any tangible real-world stimuli or categories. This would allow us to test if the brain can learn any arbitrary boundary in order to accumulate rewards. In light of previous work with BCI, we suspect it can, albeit slowly.
