# Supplementary material for: Unconscious reinforcement learning of hidden brain states supported by confidence
Source: Nat Commun. 2020 Aug 31;11:4429. doi: 10.1038/s41467-020-17828-8 (PMC7459278; doi:10.1038/s41467-020-17828-8)
Supplement: Supplementary file 3 — Reporting Summary [file 41467_2020_17828_MOESM3_ESM.pdf]

nature research

Corresponding author(s): Aurelio Cortese

Last updated by author(s): Jun 15, 2020

Reporting Summary

Nature Research wishes to improve the reproducibility of the work that we publish. This form provides structure for consistency and transparency in reporting. For further information on Nature Research policies, see Authors & Referees and the Editorial Policy Checklist.

Statistics

For all statistical analyses, confirm that the following items are present in the figure legend, table legend, main text, or Methods section.

n/a	Confirmed	
☐	☒	The exact sample size (n) for each experimental group/condition, given as a discrete number and unit of measurement
☐	☒	A statement on whether measurements were taken from distinct samples or whether the same sample was measured repeatedly
☐	☒	The statistical test(s) used AND whether they are one- or two-sided<br>*Only common tests should be described solely by name; describe more complex techniques in the Methods section.*
☐	☒	A description of all covariates tested
☐	☒	A description of any assumptions or corrections, such as tests of normality and adjustment for multiple comparisons
☐	☒	A full description of the statistical parameters including central tendency (e.g. means) or other basic estimates (e.g. regression coefficient) AND variation (e.g. standard deviation) or associated estimates of uncertainty (e.g. confidence intervals)
☐	☒	For null hypothesis testing, the test statistic (e.g. F, t, r) with confidence intervals, effect sizes, degrees of freedom and P value noted<br>*Give P values as exact values whenever suitable.*
☒	☐	For Bayesian analysis, information on the choice of priors and Markov chain Monte Carlo settings
☐	☒	For hierarchical and complex designs, identification of the appropriate level for tests and full reporting of outcomes
☐	☒	Estimates of effect sizes (e.g. Cohen's d, Pearson's r), indicating how they were calculated

Our web collection on statistics for biologists contains articles on many of the points above.

Software and code

Policy information about availability of computer code

Data collection	MATLAB R2011b
Data analysis	MRIcroGL (https://www.nitrc.org/projects/mricron), Matlab r2011b, r2016b (https://jp.mathworks.com/products/matlab.html), mrVista (http://vistalab.stanford.edu/software/), SLR toolbox v1.51 (https://bicr.atr.jp//~oyamashi/SLR_WEB.html), SPM12 (https://www.fil.ion.ucl.ac.uk/spm/), CONN toolbox v.17 (https://web.conn-toolbox.org), Freesurfer (http://surfer.nmr.mgh.harvard.edu/), TurboBrain Voyager v4 (Brain Innovation, https://www.brainvoyager.com/)

For manuscripts utilizing custom algorithms or software that are central to the research but not yet described in published literature, software must be made available to editors/reviewers. We strongly encourage code deposition in a community repository (e.g. GitHub). See the Nature Research guidelines for submitting code & software for further information.

Data

Policy information about availability of data

All manuscripts must include a data availability statement. This statement should provide the following information, where applicable:

- Accession codes, unique identifiers, or web links for publicly available datasets
- A list of figures that have associated raw data
- A description of any restrictions on data availability

The preprocessed source data and codes used to produce the figures and results presented here will be made available at the time of publication at https://bicr.atr.jp/decnefpro/. Access to the database will be provided upon reception of a signed application form, agreeing to the terms of use (request from a principal investigator, data used only for legitimate scientific research).

Field-specific reporting

Please select the one below that is the best fit for your research. If you are not sure, read the appropriate sections before making your selection.

☒ Life sciences ☐ Behavioural & social sciences ☐ Ecological, evolutionary & environmental sciences

For a reference copy of the document with all sections, see nature.com/documents/nr-reporting-summary-flat.pdf

Life sciences study design

All studies must disclose on these points even when the disclosure is negative.

Sample size	No statistical methods were used to calculate the sample size. Sample size was determined based on previous studies using real-time fMRI paradigms with multiple sessions.
Data exclusions	22 participants were initially recruited. One subject was removed because of corrupted data; one subject withdrew from the experiment after the first stage. 20 subjects were selected for the second stage (online gambling task), of which one was removed after the first scanning session due to a technical issue (scanner misalignment between initial and new sessions), while a second subject was removed due to a bias issue with online decoding (all outputs were strictly of the same class). For few participants 1 (3 in one case) block of trials (12 trials) had to be removed due to temporary issues during the experiment, e.g. in spatial alignment compromising the validity of the information fed back to participants.
Replication	Analyses were performed on the population level, across individuals, and the variation in subject responses was incorporated into statistical modeling. The variability in response between individuals is also shown through the plotting of individual raw data or data ranges in the figures as applicable.
Randomization	Allocation was not random in order to prevent the potential confound of large differences in decoding accuracies between groups. Such a large difference would have made the interpretation of the results very difficult. Therefore, participants were assigned to a given group with the specific goal as to minimize the difference in decoding accuracy between the groups.
Blinding	Blinding was not relevant to this study because task conditions were defined in real-time through machine learning -based decoding of brain activity patterns. The experimenter thus had no control over the sequence of trials.

Reporting for specific materials, systems and methods

We require information from authors about some types of materials, experimental systems and methods used in many studies. Here, indicate whether each material, system or method listed is relevant to your study. If you are not sure if a list item applies to your research, read the appropriate section before selecting a response.

Materials & experimental systems

n/a	Involved in the study	
☒	☐	Antibodies
☒	☐	Eukaryotic cell lines
☒	☐	Palaeontology
☒	☐	Animals and other organisms
☐	☒	Human research participants
☒	☐	Clinical data

Methods

n/a	Involved in the study	
☒	☐	ChIP-seq
☒	☐	Flow cytometry
☐	☒	MRI-based neuroimaging

Human research participants

Policy information about studies involving human research participants

Population characteristics	22 participants (23.6 ± 4.0 y.o.; 5 females) with normal or corrected-to-normal vision participated in the first stage of the experiment
Recruitment	Participants were recruited locally in the Kyoto and Nara areas, through a dedicated office at the institution (experimenter was not involved in recruitment). Participants were students in local universities as well as working professionals.
Ethics oversight	Institutional Review Board of ATR (Advanced Telecommunication Research Institute)

Note that full information on the approval of the study protocol must also be provided in the manuscript.

Magnetic resonance imaging

Experimental design

Design type	Real-time event-related, resting-state
Design specifications	session 0: 10 blocks<br>session 1-3: 3 sessions over 3 days, on each day with a maximum of 12 blocks (depending on available time). Before the beginning of the task session, participants underwent a 5 minutes resting-state scan.<br>In both cases each trial contained a 6 secs inter-trial interval between last and next event.
Behavioral performance measures	variables recorded: perceptual choice, decision confidence, gambling action<br>to evaluate whether task was performed as expected we used a combination of mean + standard deviation as well as computational learning models.

Acquisition

Imaging type(s)	functional
Field strength	3T
Sequence & imaging parameters	Gradient T2*-weighted EPI (echoplanar) functional images with blood-oxygen-level-dependent (BOLD) sensitive contrast and multi-band acceleration factor 6 were acquired. 72 contiguous slices (TR = 1 sec, TE = 30 ms, flip angle = 60 deg, voxel size = 2×2×2 mm3, 0 mm slice gap) oriented parallel to the AC-PC plane were acquired, covering the entire brain. T1-weighted images (MP-RAGE; 256 slices, TR = 2 s, TE = 26 ms, flip angle = 80 deg, voxel size = 1×1×1 mm3, 0 mm slice gap) were also acquired at the end of stage 1. The scanner was realigned to subjects' head orientations with the same parameters on all sessions.
Area of acquisition	whole brain

Diffusion MRI ☐ Used ☒ Not used

Preprocessing

Preprocessing software	preprocessing for decoding session 0):<br>BOLD signals in native space were preprocessed in MATLAB Version 7.13 (R2011b) with the mrVista software package for MATLAB. The mrVista package uses functions from SPM12. All functional images underwent 3D motion correction. No spatial or temporal smoothing was applied. Rigid-body transformations were performed to align the functional images to the structural image for each subject. A grey-matter mask was used to extract fMRI data only from grey-matter voxels for further analyses. Regions of interest (ROIs) were anatomically defined through cortical reconstruction and volumetric segmentation using the Freesurfer software and for visual cortex through a probabilistic map atlas (Wang et al, Cereb Cortex 2015).<br><br>real-time preprocessing of event-related data session 1-3):<br>Measured whole-brain functional images underwent 3D motion correction using Turbo BrainVoyager 4. The rest of the preprocessing was performed with Matlab r2011b. In each block, the initial 10 sec of fMRI data were discarded to avoid unsaturated T1 effects. First, measured whole-brain functional images underwent 3D motion correction. Second, time-courses of BOLD signal intensities were extracted from each of the voxels identified in the decoder analysis for the target ROI. Third, the time-course was detrended (removal of linear trend), and z-score normalized for each voxel using BOLD signal intensities measured up to the last time point.<br><br>offline preprocessing of event-related data session 1-3):<br>Image analysis was performed with SPM12. Raw functional images underwent realignment to the first image of each session. Structural images were re-registered to mean EPI images and segmented into grey and white matter. The segmentation parameters were then used to normalize and bias-correct the functional images. Normalized images were smoothed using a Gaussian kernel of 7 mm full-width at half-maximum.<br>Onset regressors at the beginning of outcome presentation (reward feedback) were modulated by a parametric regressor, trial-by-trial prediction error from computational modeling. Other regressors of no interest included onset regressors for each trial event (RDM, choice, confidence, action selection, reward outcome), 6 motion regressors and block regressors. Adding a reward regressor meant that the signal correlating with prediction error was not confounded by mere reward.<br><br>preprocessing of resting state session 1-3):<br>Using the CONN toolbox resting state data underwent realignment and unwarping, centred to (0,0,0) coordinates, slice-timing correction, outlier detection, smoothing and finally denoising.
Normalization	Spatial normalization employed a deformation field estimation, as a 3-dimensional nonrigid transformation model
Normalization template	MNI152 normalization
Noise and artifact removal	All functional images underwent 3D motion correction. Time-courses were detrended for removal of low-frequency drifts.

Volume censoring	resting-state data: CONN toolbox, removing outliers with >2std in search volume

Statistical modeling & inference

Model type and settings	multivariate, mass univariate
Effect(s) tested	We tested for motion direction, confidence, prediction error representations in activity patterns from predefined ROIs. We also tested for parametric modulation of brain activity by a computational model-derived variable (signed prediction error). Finally, we tested for positive increase or decrease in connectivity between a seed region in the basal ganglia defined through parametric modulation analysis on independent data (session 3)

Specify type of analysis: ☐ Whole brain ☐ ROI-based ☒ Both

Anatomical location(s): dorsolateral prefrontal cortex, visual cortex, basal ganglia

Statistic type for inference (See Eklund et al. 2016)	cluster-level
Correction	FPR, FDR

Models & analysis

n/a	Involved in the study
☐	☒ Functional and/or effective connectivity
☒	☐ Graph analysis
☐	☒ Multivariate modeling or predictive analysis

Functional and/or effective connectivity	Pearson correlation
Multivariate modeling and predictive analysis	Models were trained and tested using k-fold cross-validation. Trials were divided in k folds, at each iteration one fold was left out as test set. Final accuracy was taken as the average of all test folds accuracies. Feature selection was done using automatic-relevance determination, with sparsity prior (similar to L1-norm regularization).
